# Protective Effect of N-Acetylcysteine against Oxidative Stress Induced by Zearalenone via Mitochondrial Apoptosis Pathway in SIEC02 Cells

**DOI:** 10.3390/toxins10100407

**Published:** 2018-10-09

**Authors:** Jingjing Wang, Mengmeng Li, Wei Zhang, Aixin Gu, Jiawen Dong, Jianping Li, Anshan Shan

**Affiliations:** Institute of Animal Nutrition, Northeast Agricultural University, Harbin 150030, China; JF_JING@hotmail.com (J.W.); limengmeng@outlook.com (M.L.); zhangwei910315@hotmail.com (W.Z.); aixingu@hotmail.com (A.G.); dongjiawen2018@outlook.com (J.D.)

**Keywords:** Zearalenone, N-acetylcysteine, SIEC02 cells, Mitochondrial apoptosis

## Abstract

Zearalenone (ZEN), a nonsteroidal estrogen mycotoxin, is widely found in feed and foodstuffs. Intestinal cells may become the primary target of toxin attack after ingesting food containing ZEN. Porcine small intestinal epithelial (SIEC02) cells were selected to assess the effect of ZEN exposure on the intestine. Cells were exposed to ZEN (20 µg/mL) or pretreated with (81, 162, and 324 µg/mL) N-acetylcysteine (NAC) prior to ZEN treatment. Results indicated that the activities of glutathione peroxidase (Gpx) and glutathione reductase (GR) were reduced by ZEN, which induced reactive oxygen species (ROS) and malondialdehyde (MDA) production. Moreover, these activities increased apoptosis and mitochondrial membrane potential (ΔΨm), and regulated the messenger RNA (mRNA) expression of Bax, Bcl-2, caspase-3, caspase-9, and cytochrome c (cyto c). Additionally, NAC pretreatment reduced the oxidative damage and inhibited the apoptosis induced by ZEN. It can be concluded that ZEN-induced oxidative stress and damage may further induce mitochondrial apoptosis, and pretreatment of NAC can degrade this damage to some extent.

## 1. Introduction

Zearalenone (ZEN), a secondary metabolite produced by fungi of the genus *Fusarium*, can seriously affect animal growth, reproduction, and immune function [1,2,3]. Studies estimate that one-fourth of the global food and feed output is contaminated by mycotoxins, and the number is likely to be closer to 50% if new fungal toxins are considered for limited data [4,5]. ZEN is being increasingly recognized as a frequent contaminant in animal feeding, which has a significant effect on human and animal health [6,7]. According to a recent report, the United States Department of Agriculture (USDA), the European Food Safety Authority (EFSA), and China are concerned about mycotoxin contamination in cereals, and they have renewed the maximum residue limits (MRLs) in food and feeds [8]. The MRLs in food is 350 µg/kg, 350 µg/kg, and 60 µg/kg, respectively. EFSA and China also reiterated that the MRLs in feed is 2000 µg/kg and 500 µg/kg [8].

It is shown that most animals have a certain sensitivity to ZEN, and the pig is the most sensitive animal [9]. The primary attack target of toxins from ingested food or feed is the gastrointestinal tract, and thus it affects intestinal function [10,11]. The main metabolites of ZEN are α-zearalenol (α-ZOL) and β-zearalenol (β-ZOL) [12]. Establishing a barrier model (IPEC-1) in vitro, study has shown that ZEN and its metabolites damage barrier function by reducing the immune response [13]. It has been reported that ZEN affects the villous structures and reduces the expression of junction proteins in a dose-dependent manner in pregnant rats [14]. The estrogen-like effects of ZEN and its derivatives have been determined in vivo and in vitro [13,15]. A further experiment has demonstrated that oxidative damage is likely to be evoked as one of the main pathways of ZEN toxicity [16]. Moreover, data suggested that ZEN induced apoptosis in a dose-dependent manner in many cell lines (HepG2, porcine granulosa, and SHSY-5Y cells) [6,17,18]. Therefore, it is necessary to analyze the effect of ZEN on the intestinal tract.

N-acetylcysteine (NAC), the precursor of glutathione, is a water-soluble molecule that has antioxidant, anti-inflammatory, and tumor-inhibitory properties [19,20]. A previous study has shown that NAC can exert antioxidant effects both in vitro and in vivo [21]. It is generally assumed that the action of NAC is to scavenge free radicals by increasing the intracellular glutathione (GSH) level [22]. More recently, studies have found that NAC can directly inhibit the production of reactive oxygen species (ROS), and thus inhibit apoptosis [23,24]. Similarly, NAC pretreatment can reduce lipid peroxidation and inhibit apoptosis [25]. In addition, NAC also plays an important role in protecting renal function by reducing oxidative stress [26,27].

Cells were poisoned by ZEN, cells may undergo complex and different pathways of damage, including apoptosis [16,18]. The SIEC02 cells retained the morphological and functional characteristics that were typical of primary swine intestinal epithelial cells by introducing the human telomerase reverse transcriptase, and thus they provide a cell model in vitro [28]. However, the mechanism underlying the apoptosis of intestinal cells after exposure to ZEN is still not totally understood. Therefore, the purpose of this study was to evaluate whether ZEN-induced oxidative damage could further lead to apoptosis, and whether the addition of NAC could alleviate this negative effect on SIEC02 cells.

## 2. Results

### 2.1. Effects of ZEN and NAC on Cell Viability

**Figure 1:**
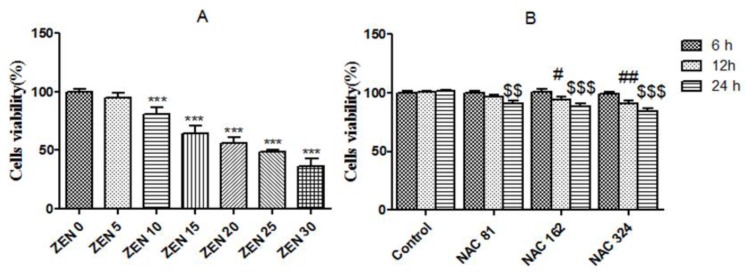
Effects of zearalenone (ZEN) and N-acetylcysteine (NAC) on SIEC02 cells viability. Cells were treated without or with different concentrations of ZEN (0, 5, 10, 15, 20, 25 and 30 µg/mL) for 24 h (**A**). Cells were pretreated without or with different concentrations of NAC (81, 162 and 324 µg/mL) for 6 h, 12 h, and 24 h (**B**). Cells survival was measured by Cell Counting Kit-8 (CCK-8) assay. The values are mean ± SD of three independent experiments. *** indicates a significant difference between ZEN and control at *P* < 0.001. #, ## indicates a significant difference of 12 h between NAC and control, with significant differences at *P* < 0.05 and *P* < 0.01. $$, $$$ indicates a significant difference of 24 h between NAC and the control at *P* < 0.01 and *P* < 0.001.

To examine the cytotoxic effects of ZEN, SIEC02 cells were incubated with ZEN (5, 10, 15, 20, 25 and 30 µg/mL) for 24 h. After incubation, ZEN treatment inhibited the cell viability markedly in a dose-dependent manner (Figure 1A). Cell viability was only 36.30% at a ZEN concentration of 30 µg/mL, and the inhibitory concentration of IC_50_ (50% inhibitory concentration) was 22.68 ± 0.80 µg/mL. Hence, a cytotoxic concentration of ZEN (20 µg/mL) was selected for subsequent experiments. In addition, NAC alone did not show any cytotoxicity at concentrations (81, 162, and 324 µg/mL) of incubation for 6 h. However, compared with the control group, cell viability was significantly reduced when cells were treated by NAC (162 and 324 µg/mL) for 12 h (*P* < 0.05). All three concentrations of NAC significantly reduced cell viability for 24 h (*P* < 0.01) (Figure 1B). Based on the additions shown in Figure 1B, NAC concentrations (81, 162 and 324 µg/mL) were selected for pretreating the cells for 6 h prior to the ZEN treatment.

### 2.2. Effects of ZEN and NAC on Oxidative Stress

#### 2.2.1. Glutathione peroxidase (Gpx) Activity

**Figure 2:**
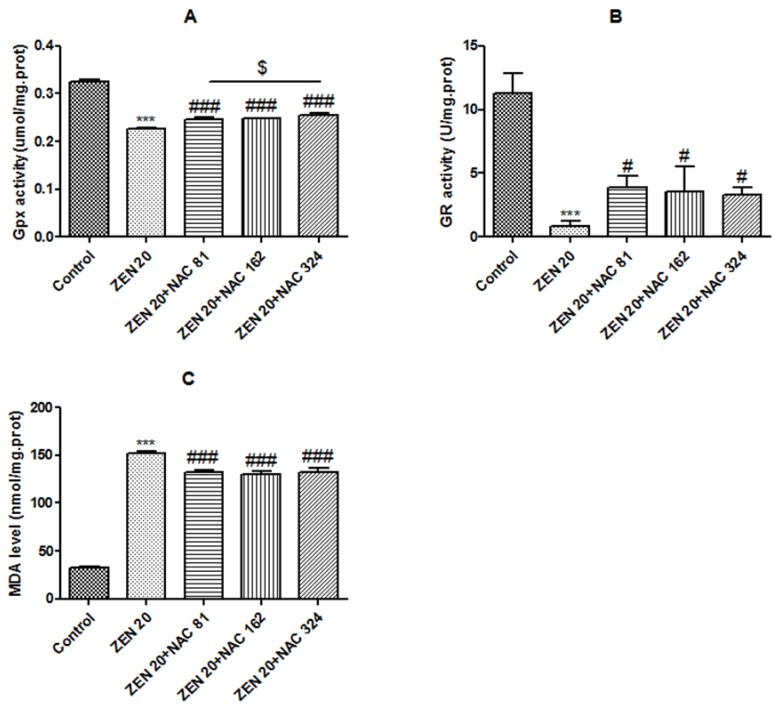
Effect of ZEN (20 µg/mL) and NAC (81, 162 and 324 µg/mL) on intracellular glutathione peroxidase (Gpx), glutathione reductase (GR) activity, and malondialdehyde (MDA) levels. Cells were exposed to ZEN for 24 h, including NAC pretreatment for 6 h. The results of Gpx, GR, and MDA were µmol/mg, U/mg, nmol/mg of protein, respectively. Each set of data shows the mean ± SD of three independent experiments. *** indicates a significant difference between ZEN and control *P* < 0.001. #, ###, indicates a significant difference between ZEN and NAC in mutual treatment at *P* < 0.05 and *P* < 0.001. $ indicates a significant difference between three concentrations of NAC at *P* < 0.05 (**A**–**C**).

Data on the activity of antioxidative enzymes and related products in SIEC02 cells is summarized in Figure 2. As shown in Figure 2A, Gpx activity was significantly reduced after ZEN treatment on 0.227 µmol/mg of protein, compared with the control group (0.325 µmol/mg) (*P* < 0.001). The Gpx activity was restored to a certain extend by the pretreatment of cells with NAC (81, 162 and 324 µg/mL) (*P* < 0.001) and increased to 0.247, 0.248 and 0.254 µmol/mg of protein, respectively. Based on these data, NAC pretreatment could significantly increase the reduction in Gpx activity induced by ZEN, and the optimal concentration of NAC was 324 µg/mL (*P* < 0.05).

#### 2.2.2. Glutathione reductase (GR) Activity

According to Figure 2B, compared with the control group (11.307 U/mg), the GR activity of ZEN treatment was significantly reduced to 0.857 U/mg of protein (*P* < 0.001). The reduction in GR activity induced by ZEN was restored to a certain extend by the treatment of cells with NAC (81, 162 and 324 µg/mL) (*P* < 0.05) and increased to 3.859, 3.537 and 3.269 U/mg of protein, respectively. Based on these data, NAC pretreatment could significantly increase the activity of GR. Three concentrations of NAC did not reach a significant level.

#### 2.2.3. Malondialdehyde (MDA) Level

As shown in Figure 2C, the MDA level of ZEN treatment was significantly higher (151.9 nmol/mg of protein) than the control group (32.2 nmol/mg) (*P* < 0.001). Pretreatment with NAC (81, 162 and 324 µg/mL) significantly reduced the increase in the MDA level as induced by ZEN (*P* < 0.001), and decreased to 132.2, 130.2 and 132.5 nmol/mg of protein, respectively. Three concentrations of NAC did not reach a significant level.

### 2.3. Effects of ZEN and NAC on Intracellular ROS Generation

**Figure 3:**
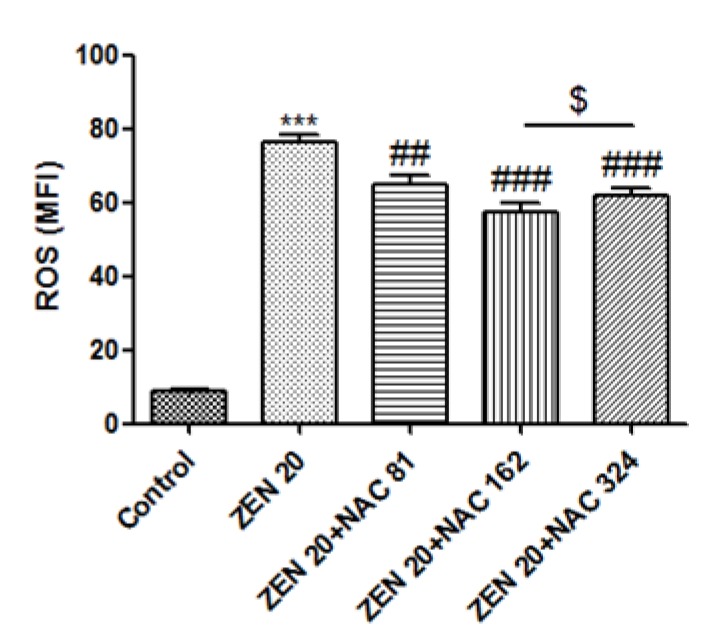
Effect of ZEN (20 µg/mL) and NAC (81, 162 and 324 µg/mL) on intracellular reactive oxygen species (ROS) production. Cells were exposed to ZEN for 24 h, including NAC pretreatment for 6 h. The results are expressed as mean fluorescent intensity (MFI). Each set of data shows the mean ± SD of the three independent experiments. *** indicates a significant difference between ZEN and control at *P* < 0.001. ##, ### indicates a significant difference between ZEN and NAC in mutual treatment at *P* < 0.01 and *P* < 0.001. $ indicates a significant difference between three concentrations of NAC at *P* < 0.05.

ROS was generated as by-products of cellular metabolism, which could trigger oxidative stress at high concentrations [29] . ROS production was monitored by measuring the fluorescence intensity of DCFH-DA (2',7'-dichlorodihydrofluorescein diacetate dye). DCFH-DA is a fluorescent probe of ROS. The non-fluorescent fluorescin DCFH-DA derivatives will emit fluorescence after being oxidized by the radicals generated by the toxins. In the presence of ROS, H_2_-DCF is rapidly oxidized to become highly fluorescent DCF [30,31]. The result indicated that ZEN could significantly induce ROS accumulation in SIEC02 cells (*P* < 0.001) (Figure 3) and the ROS content in the ZEN group was significantly higher than that in control group. As observed in Figure 3, NAC pretreatment significantly reduced ZEN-induced ROS production (*P* < 0.01), and the optimal concentration of NAC was 162 µg/mL (*P* < 0.05).

### 2.4. Effects of ZEN and NAC on Apoptosis

**Figure 4:**
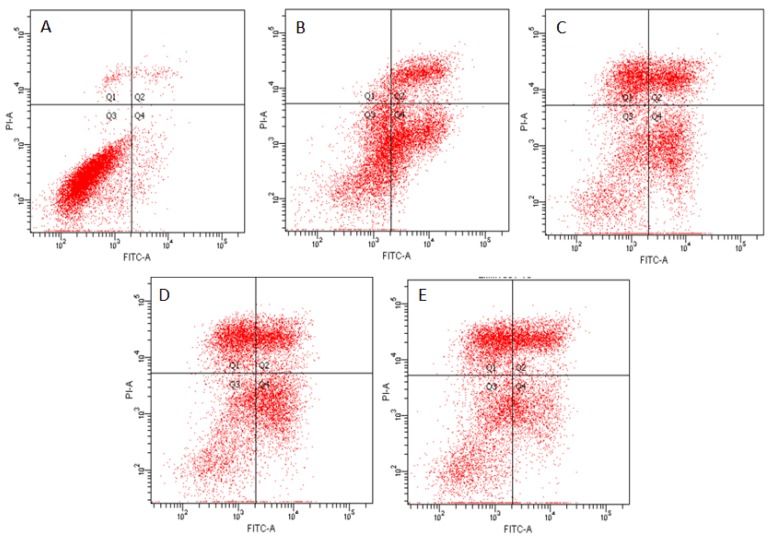

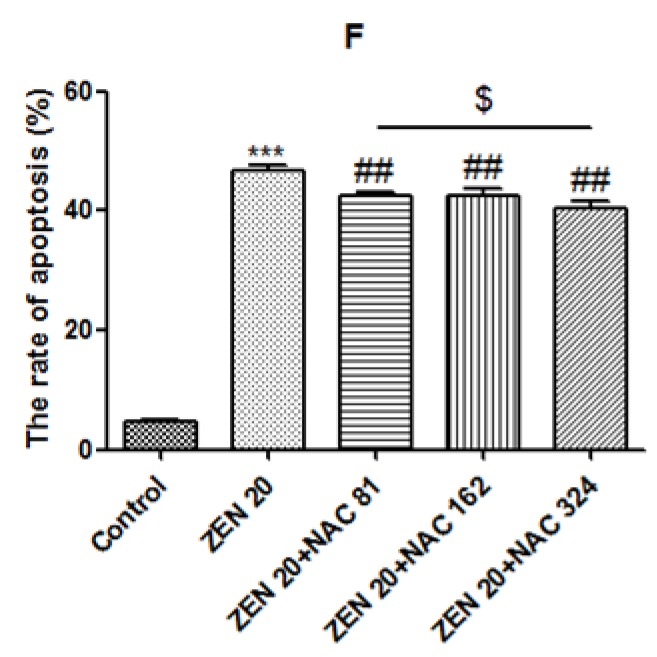
Annexin V-FITC/PI flow cytometry was used to detect SIEC02 cells treated with ZEN (20 µg/mL) and NAC (81, 162 and 324 µg/mL). The Q1, Q2, Q3, and Q4 gates, respectively, represented dead cells, the late stage of cell apoptosis, normal cells, and the early stage of cell apoptosis (**A**, **B**, **C**, **D** and **E** are control, ZEN 20 µg/mL, ZEN 20 µg/mL + NAC 81 µg/mL, ZEN 20 µg/mL + NAC 162 µg/mL, and ZEN 20 µg/mL + NAC 324 µg/mL, respectively). Apoptosis results are expressed as the rate of apoptosis. Each set of data shows the mean ± SD of the three independent experiments. *** indicates a significant difference between ZEN and control at *P* < 0.001. ## indicates a significant difference between ZEN and NAC in mutual treatment at *P* < 0.01. $ indicates a significant difference between three concentrations of NAC at *P* < 0.05 (**F**).

Cells were stained with Annexin V-FITC/PI to determine whether ZEN induced cell apoptosis and its consequences following alleviation with NAC were evaluated. Compared with the control group (Figure 4A), the number of living cells were decreased and the early apoptosis of ZEN treatment, and the late apoptotic cells were significantly increased (*P* < 0.05) (Figure 4B and 4F). As seen in Figure 4C,D,E and F, the apoptotic cells of Q2 and Q4 were reduced compared with Figure 4B, and the apoptotic rate was also significantly reduced (*P* < 0.01) by NAC pretreatment. The optimal concentration of NAC was 324 µg/mL (*P* < 0.05) (Figure 4F).

### 2.5. Effects of ZEN and NAC on the Change of ΔΨm

**Figure 5:**
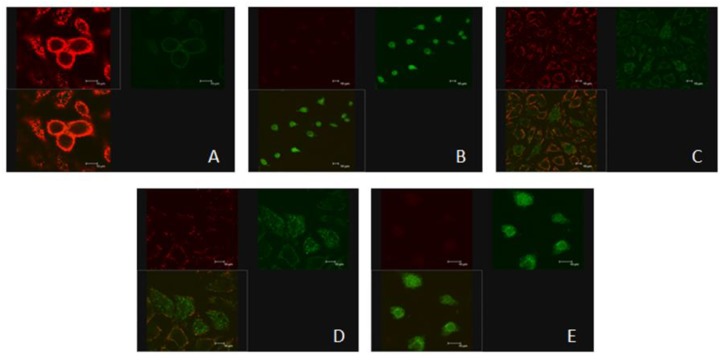

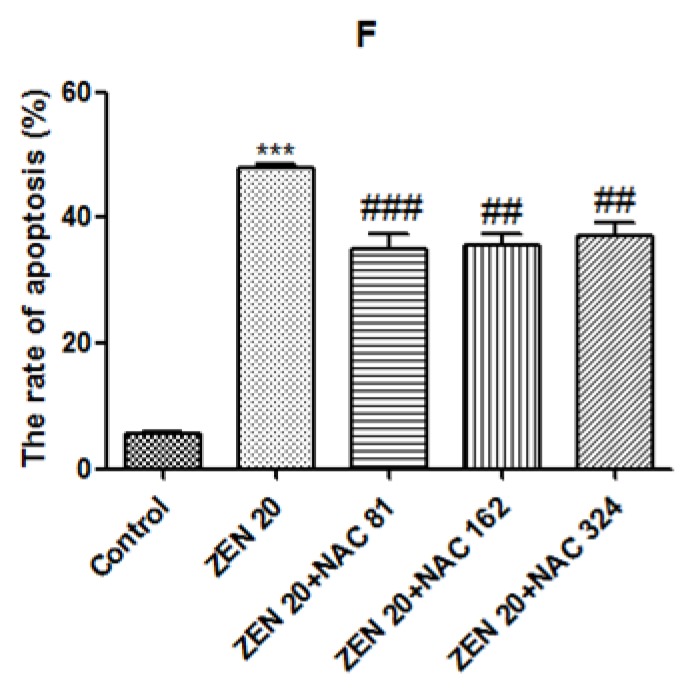
A laser scanning confocal microscope was used to observe the changes of mitochondrial membrane potential (ΔΨm) in SIEC02 cells treated with ZEN and NAC. The scanning pictures were as shown in the figure (**A**–**E** are control, ZEN 20 µg/mL, ZEN 20 µg/mL + NAC 81 µg/mL, ZEN 20 µg/mL + NAC 162 µg/mL and ZEN 20 µg/mL + NAC 324 µg/mL, respectively). Scale bar: 10 µm. The results are expressed as apoptosis rate (**F**); each set of data shows the mean ± SD of the three independent experiments. *** indicates a significant difference between ZEN and control at *P* < 0.001. ##, ### indicates a significant difference between ZEN and NAC in mutual treatment at *P* < 0.01 and *P* < 0.001 (**F**).

JC-1 (mitochondrial probe) formed J-aggregates in the mitochondrial matrix (red) at high ΔΨm in nonapoptotic cells, and formed monomeric (green) at low ΔΨm in apoptotic cells (Figure 5). Therefore, the change of ΔΨm was reflected by decreasing the red and green fluorescence ratio. The effects of different treatments on mitochondrial membrane potential in SIEC02 cells are shown in Figure 5A–E. As shown in Figure 5B, the green aggregates were increased compared with the control group (Figure 5A), indicating that the ΔΨm was decreased, the cell membrane was severely damaged, and the apoptosis rate was significantly increased (*P* < 0.001) (Figure 5F). After NAC pretreatment, green aggregates showed signs of weakening (Figure 5C,D and E) compared with the ZEN group (Figure 5B). The apoptosis rate of the cells was significantly decreased by NAC pretreatment (*P* < 0.01) and it did not reach a significant level in the three concentrations of NAC (Figure 5F).

### 2.6. Effects of ZEN and NAC on Apoptosis-Related mRNA Expression

#### 2.6.1. Bax

**Figure 6:**
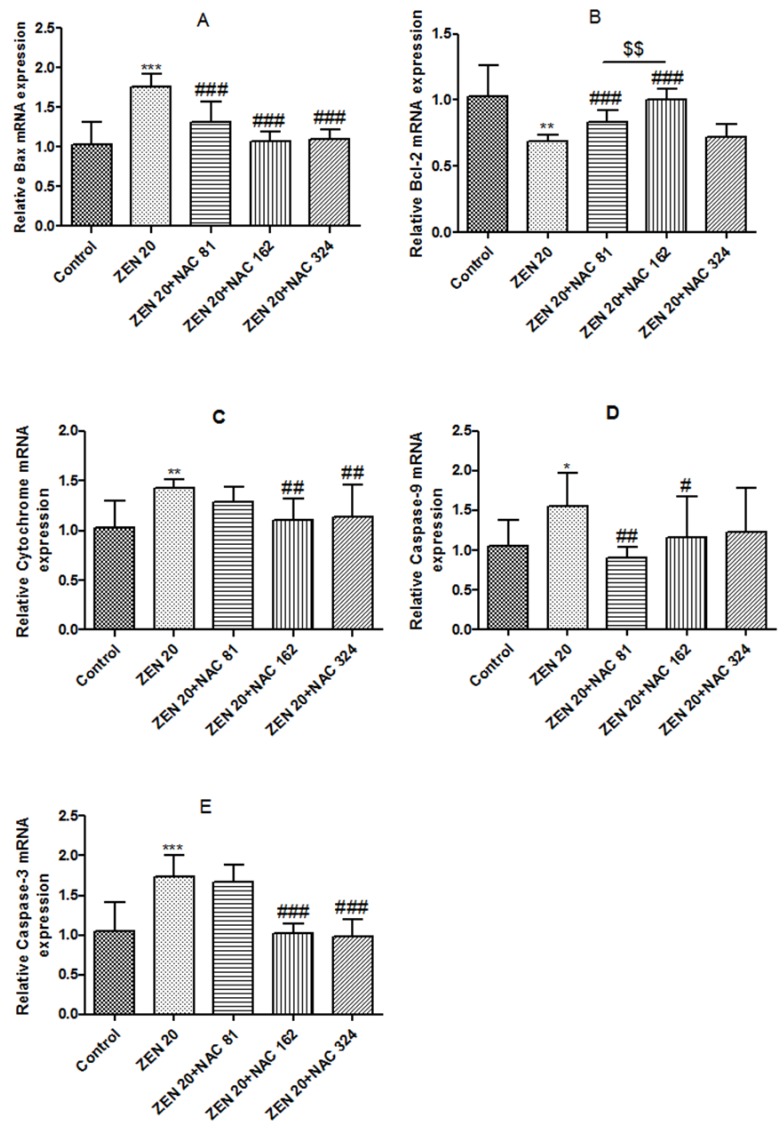
The results of the ZEN (20 µg/mL) and NAC (81, 162 and 324 µg/mL) expression levels of each apoptotic gene (Bcl-2, Bax, cytochrome c, caspase-9, caspase-3) in the SIEC02 cells. Cells were exposed to ZEN for 24 h, including NAC pretreatment for 6 h. The results are expressed relative to the expression of actin; each set of data shows the mean ± SD of the three independent experiments. *, **, *** indicates a significant difference between ZEN and control at *P* < 0.05, *P* < 0.01, and *P* < 0.001. #, ##, ### indicates a significant difference between ZEN and NAC in mutual treatment at *P* < 0.05, *P* < 0.01, and *P* < 0.001. $$ indicates a significant difference between three concentrations of NAC at *P* < 0.01 (**A**–**E**).

The effects of ZEN and NAC protection apoptosis-related mRNA levels via caspase pathways are shown in Figure 6. As shown in Figure 6A, the mRNA expression levels of Bax were significantly increased by ZEN, compared with the control group (*P* < 0.001). Compared with the ZEN treatment, the NAC-pretreated significantly reduced the mRNA expression of Bax (*P* < 0.001). Three concentrations of NAC did not reach a significant level.

#### 2.6.2. Bcl-2

The expression of Bcl-2 mRNA was opposite to that of Bax. As shown in Figure 6B, the mRNA expression levels of Bax were significantly decreased in the ZEN group (*P* < 0.01). Compared with ZEN treatment, the production of Bcl-2 could not be significantly decreased at a NAC concentration of 324 µg/mL. When the concentration of NAC were 81 and 162 µg/mL, the mRNA expression of Bcl-2 significantly increased (*P* < 0.001), and the optimal concentration of NAC was 162 µg/mL (*P* < 0.01).

#### 2.6.3. *Cytochrome* c (Cyto c)

As shown in Figure 6C, the mRNA expression levels of cyto c were significantly increased by ZEN (*P* < 0.01). Compared with ZEN treatment, the production of cyto c could not be significantly decreased at a NAC concentration of 81 µg/mL. When the NAC concentration of NAC were 162 and 324 µg/mL, cyto-c production was significantly decreased (*P* < 0.01).

#### 2.6.4. Caspase-9 and Caspase-3 

Similar to cyto c mRNA expression, caspase-9 and caspase-3 mRNA levels were significantly increased by ZEN (*P* < 0.05) (Figure 6D,E). Compared with ZEN treatment, the mRNA expression of caspase-9 was significantly reduced at the NAC concentrations of 81 and 162 µg/mL (*P* < 0.05). At the same time, when the NAC concentration of NAC were 162 and 324 µg/mL, the mRNA expression of caspase-3 was significantly increased (*P* < 0.001).

## 3. Discussion

ZEN and its major metabolites are secondary metabolites of *Fusarium* fungi that produce cell damage [12,32]. A number of studies have found that ZEN has an inhibitory effect on cell proliferation [15,16]. The most direct indicator is the number of living cells due to toxic changes. The IC_50_ is an important indicator of the cellular response to chemical effects. In human hepatoma cells (HepG2) and colorectal cancer cells (HCT116), the IC_50_ is 100 µM and 60 µM, respectively, and cell proliferation undergoes a dose-dependent reduction after ZEN treatment [18,33]. In this experiment, Cell Counting Kit-8 (CCK-8) method was used to detect the effect of ZEN on the activity of SIEC02 cells cultured in vitro. The results showed that after treatment with ZEN for 24 h, the IC_50_ range was 22.68 ± 0.80 µg/mL (62.89 µM). This value might differ slightly from that just mentioned, because of differences in cell lines. With the increase in ZEN concentration, the cell activity gradually decreased and showed a dose-response relationship.

The gastrointestinal tract is the primary site of toxin interaction, and intestinal epithelial cells are an important first target site for mycotoxin [34,35]. Most studies of toxicity have shown that the cytotoxicity of ZEN is determined by the production of ROS, DNA damage, and an increased formation of lipid peroxidation [36,37]. In normal physiological processes, small amounts of ROS are generated as by-products of cellular metabolism, primarily in the mitochondria [29,32]. ROS are highly reactive molecules, among which oxygen-free radicals can destroy cell structures, and increase lipid peroxidation of biofilms [38,39]. MDA, as the final product of lipid peroxidation, which can cause cell serious injuries in membrane structure, changes the permeability of cell membranes, leading to cell apoptosis, and necrosis [40]. Therefore, the measurement of MDA content can indirectly reflect the degree of lipid peroxidation. In this experiment, our results showed that 20 µg/mL of ZEN significantly increased ROS and MDA contents in SIEC02 cells. Previous studies have found similar effects in different cell types that are exposed to ZEN. These results were consistent with the findings that ZEN can significantly increase ROS in CHO-K1 and IPEC-J2 cells and MDA in Caco-2 cells [36,41,42].

NAC, a precursor of GSH that is a free radical scavenger, which plays an important role as an antagonistic foreign chemical to the oxidative damage caused by biological organisms [22,43]. The antioxidant activity of NAC is evaluated by the enzymatic system. The antioxidant enzymes and other regulatory enzymes can be used as the first line of defense, and they play a role in scavenging free radical damage intracellularly and extracellularly [38]. GR and Gpx are the intrinsic anti-oxidative enzymes of cells [44]. Gpx catalyzes GSH to decompose H_2_O_2_ into water, and to form Glutathione oxidized (GSSG), and the GR enzyme can reduce GSSG to GSH, to scavenge excess H_2_O_2_ [45,46]. NAC could increase intracellular glutathione and produce sulfhydryl groups that directly eliminate ROS, such as hypochlorous acid, hydrogen peroxide, superoxide, and hydroxyl radicals [47,48]. Consequently, NAC increases the activity of the antioxidant enzymes Gpx and GR by restoring intracellular GSH content, thereby eliminating ROS. In this experiment, our data clearly demonstrated that the activities of Gpx and GR were significantly increased after NAC pretreatment, and that ROS was significantly reduced. Our study showed that NAC preconditioning could alleviate the activity of antioxidant enzymes in SIEC02 cells, and this result is also consistent with another team's results on the pig kidney PK15 cells [49]. In addition, pretreatment with NAC positively reduced ROS generation, suggesting a potential antioxidant for NAC, which is conformed to reduce cellular ROS by adding NAC in Jurkat T cells [50].

Apoptosis, a form of programmed cell death, is an essential process in cell growth, reproduction, and self-adjustment and it is characterized by morphological changes such as nucleosome fracture and the formation of apoptotic bodies [51,52]. In apoptosis caused by exposure to mycotoxins, mitochondrial-mediated apoptosis is the main pathway, which is activated programmed cell death by ROS via mediated by mycotoxins [53]. The mitochondria are an important source of ROS within most mammalian cells [54]. In response to apoptotic stimuli, DNA damage and oxidative stress occur, and eventually the mitochondrial pathway is triggered [30]. The mitochondrial outer membrane is highly permeability in mitochondria [31,55]. Indeed, a previous study reported that when cells are poisoned or subjected to other stimuli, the ΔΨm decreases [56]. The ΔΨm disruption is an important trigger in managing apoptosis [57]. Studies have found that ZEN may cause mitochondrial membrane hyperpolarization over a short period of time, followed by loss of the ΔΨm [58,59]. Previous in vitro experiments demonstrate that ZEN directly causes apoptosis through the mitochondrial pathway at low concentrations. Subsequently, numerous studies show that ZEN causes intracellular ROS and MDA levels to rise and ΔΨm decreases, leading to cell apoptosis [32,60,61]. Based on these findings, to reveal whether ZEN-induced apoptosis involves ΔΨm, our study focused on detecting ΔΨm in SIEC02 cells by JC-1 fluorescence to detect changes in membrane potential. Green fluorescence is indicative of the ΔΨm decline by JC-1 staining [62,63]. Our results showed that treatment with ZEN caused an increase in ROS and loss of ΔΨm, suggesting that ZEN passed the mitochondrial-mediated pathway. In this study, the percentage of ZEN cells with green fluorescence increased by NAC. This may be because NAC is deacetylated to cysteine, rapidly oxidized, and then transported to cells, which is reduced to GSH, thereby restoring the cell state [6]. The results showed that after addition of NAC pretreatment, ΔΨm increased, inhibiting apoptosis in SIEC02 cells. This result is consistent with recent studies that have promoted apoptosis of the mitochondria-mediated pathway through ZEN [64].

When mitochondrial membrane permeability changes, cyto c is first released from the mitochondrial membrane space into the cytoplasm, resulting in subsequent activation of capase-9 [65,66]. Next, activation of caspase-9 activates the downstream performer (caspase-3), thereby triggering apoptosis in the cascade [67]. In the mitochondrial apoptosis pathway, caspase-9 is a key activator of the caspase cascade; caspase-3 is located downstream of the apoptotic ordered cascade reaction and it is the key executor of the transfer, and its activation marks the process of apoptosis [68,69]. Previous studies have found that the production of ROS can proceed from the release of cyto c [70]. Furthermore, a study has shown that ROS can further release cyto c from the mitochondria into the cytoplasm [64]. In this experiment, after ZEN exposure, both intracellular ROS and cyto c increased significantly consistent with previous studies. The Bcl-2 protein family may regulate mitochondrial permeability through both permeabilization of the mitochondrial membrane or translocation of cyto c [28]. Bax is the representative of apoptosis-promoting proteins, and Bcl-2 is a gene that inhibits apoptosis [71]. When the cell damage is serious, Bcl-2 protein expression decreases and Bax protein expression increases [72]. The current study showed that ZEN downregulated Bcl-2 and upregulated Bax mRNA expression, which triggered apoptosis in SIEC02 cells. In the current study, our results showed that after treatment with ZEN toxins, a significant increase in apoptosis was observed. ZEN treatment upregulated mRNA expression of cyto c, caspase-3, and caspase-9. According to the results of this experiment, pretreatment with NAC could inhibit the expression of proapoptotic genes in cells, increase the expression of antiapoptotic genes, and reduce the cell damage induced by ZEN.

## 4. Conclusions

In conclusion, the results of this study suggested that ZEN induced oxidative stress in SIEC02 cells and sequentially promoted apoptosis through the mitochondrial pathway. ZEN produced substantial cytotoxicity to SIEC02 cells because of elevated ROS levels, which in turn led to increased caspase-3 activation and apoptosis. In addition, NAC pretreatment increased antioxidant enzyme activity, elevated ΔΨm, and inhibited ZEN-induced apoptosis through the mitochondrial pathway. Based on these results, this study provides new insights into the mechanism of action of ZEN on intestinal cells in vitro, and the mitigation of this effect by pretreatment with NAC. However, all of the effects of ZEN and its protective mechanisms for the intestine are still not fully understood, and further research is needed.

## 5. Materials and Methods 

### 5.1. Chemicals and Reagents 

ZEN and NAC were supplied by Sigma-Aldrich (St. Louis, MO, USA). Dulbecco modified Eagle medium (DMEM)-F:12 cell culture medium was purchased from Thermo Fisher (Hyclone, Shanghai, China). Fetal bovine serum (FBS) was supplied by Gibco (Invitrogen Corporation, Grand Island, NY, USA). CCK-8 was supplied by Dojindo (Kumamoto, Japan). ROS assay kit, MDA, Gpx, and GR antioxidant kits, and ΔΨm assay kit with JC-1 were supplied by Beyotime Biotechnology (Nantong, China). Phosphate buffered saline (PBS) was purchased from Biotopped (Beijing, China). Ethanol at a concentration of 0.25% was chosen as an organic solvent for the dilution of ZEN pure product for subsequent testing.

### 5.2. Cell Culture and Conditions

SIEC02 cells were donated by Northwest A&F University. The SIEC02 cell line was derived from the mid-jejunum of neonatal, un-suckled, and 1-day-old Landrace piglets [73]. Cells (passages 15–30) were grown in DMEM-F:12 supplemented with 10% fetal calf serum, and 1% penicillin and streptomycin, and cultured at 37 °C in an atmosphere of 5% CO_2_ in a cell incubator. The cells were inoculated in a 25 cm^2^ cell culture flask (Nest, Eimer Biotechnology Company, Wuxi, China), and the culture medium was changed after 24 h. When the cells were confluent to approximately 80%–90%, the cell monolayer was washed with PBS and digested with 1× trypsin/EDTA (Beyotime Institute of Biotechnology, Nantong, China) for subsequent testing.

### 5.3. Cell Viability Assay 

The cytotoxicity of ZEN on SIEC02 cells was measured by CCK-8. Cells (0.5–0.8 × 10^6^/mL) were seeded in 96-well culture plates for 24 h (Nest, Eimer Biotechnology Company, Wuxi, China) and then treated with ZEN (0, 5, 10, 15, 20, 25 and 30 µg/mL). Similarly, cells were seeded in the same culture plates (6, 12, and 24 h) and then treated with NAC (81, 162, and 324 µg/mL). After all treatments were completed, cells were washed twice with PBS. CCK-8 was then added to a final concentration of 10% in a serum-free medium and cultured at 37 °C for 3 h. The viability rate was measured by measuring the absorbance on a microplate reader with an emission wavelength of 450 nm (SpectraMax M5, Molecular Devices, Sunnyvale, CA, USA). 

### 5.4. Experiment Design

For all experiments, cells were used at 80%–90% confluence and assigned into five treatment groups: control group (cells were incubated with FBS-DMEM-F:12 culture medium for 24 h); ZEN group (cells were incubated with 20 μg/mL ZEN for 24 h); NAC+ZEN group after cells were pretreated with NAC (81, 162 and 324 µg/mL) for 6 h, then placed with fresh culture medium containing 20 μg/mL ZEN for 24 h. The cells of the subsequent experiments (0.5–0.8 × 10^6^/mL) were seeded in 6-well culture plates (Nest, Eimer Biotechnology Company, Wuxi, China) and treated with drugs as described in this section. 

### 5.5. Determination of Antioxidant Enzyme Activity and Oxidative Products

SIEC02 cells were used for the analysis of antioxidative enzyme and related products. Briefly, cells were washed with 4 °C precooled PBS and resuspended, then lysed on ice. Antioxidant enzyme activities such as Gpx and GR were measured using the assay kit according to the manufacturer’s instructions. The results were expressed as µmol/mg or U/mg of protein, respectively. The level of MDA was measured according to the kit instructions, and the results were expressed as nmol/mg of protein. The density of each protein was detected by Enhanced BCA Protein Assay Kit (Beyotime Institute of Biotechnology, Nantong, China).

### 5.6. Detection of ROS Generation

The balance between the generation and clearance of ROS is important to maintain intracellular redox states. A flow cytometry technique was used to assess the intracellular amounts of ROS with dichlorofluorescein diacetate (DCFH-DA). Briefly, cells were washed with PBS and incubated with 10 µM DCFH-DA at 37 °C for 20 min. After incubated, cells were washed thrice with serum-free cell culture medium. Intracellular production of ROS was measured by a FACS flow cytometry (Becton-Dickinson, San Jose, CA, USA) with an excitation wavelength of 488 nm and an emission wavelength of 525 nm. The DCF fluorescence intensity indicates the amount of intracellular ROS and the results were analyzed by mean fluorescent intensity (MFI).

### 5.7. Apoptosis Detection

#### 5.7.1. Annexin V-FITC/PI Double Staining

The apoptosis of SIEC02 cells was measured by the Annexin V-FITC/PI apoptosis detection kit (Biosea Biochemicals, Shanghai, China). Briefly, cells were trypsinized and harvested via centrifugation at 2000×g and 4 °C for 5 min, then re-suspended in 500 µL binding buffer. Annexin-FITC (10 µL) was injected into the solutions and incubated for 40 min at 37 °C in the dark. Finally, 5 µL PI (10 μg/mL) was added. Scattering signals were detected by FACS flow cytometry as described in the previous section.

#### 5.7.2. ΔΨm Assay

In this experiment, ΔΨm was monitored by the cationic dye JC-1. Briefly, after treatment and washing three times with PBS, culture medium (1 mL) and JC-1 staining solution (1 mL) are added; after being fully mixed, the cells are incubated at 37 °C for 20 minutes. During incubation, according to 1 mL JC-1 staining buffer (5×) with 4 mL distilled water, JC-1 staining is mixed with JC-1 staining buffer (1×). An increase in the green/red fluorescence intensity ratio indicates mitochondrial membrane depolarization. To each well, 0.5 mL culture medium was added, and the results were detected using a laser scanning confocal microscope (LEICA, Hesse, Germany). 

#### 5.7.3. RNA Extraction and Quantitative real time polymerase chain reaction (qRT-PCR)

Total RNA was extracted using RNA fast 200 (Fastagen, Shanghai, China), according to the manufacturer's instruction. The A_260_/A_280_ ratio was measured by using a using a Nano Photometer P-Class (Implen GmbH, Munich, Germany) to ensure the purity of the RNA sample, and agarose gel electrophoresis was used to ensure the integrity of the total RNA sample. The complementary DNA (cDNA) was amplified by qRT-PCR using a SYBR Premix Ex Taq RT-PCR kit (Takara, Dalian, China) and used for RT-PCR. SYBR Green I RT-PCR kit (Takara, Dalian, China) was used to measure the mRNA expression of apoptosis-related genes (Bcl-2, Bax, cyto c, caspase-9, caspase-3), and β-actin was used as the internal control gene to correct for differences. The sequences of the specific primers used were as follows in Table 1 [49]. The sequences of the specific primers in this study used were designed from published GenBank sequences, and they were synthesized by Sangon (Shanghai, China).

RT-PCR was performed by an ABI PRISM 7500 SDS thermal cycler (Applied Biosystems, Foster City, CA, USA). The reaction was carried out using a 10 µL system of PCR, including 1.0 µL of cDNA and 0.2 μL of each primer. The cycle numbers and annealing temperature were optimized for each primer pair. The PCR cycling conditions were as follows: a denaturation step at 95 °C for 30 s, followed by 40 cycles of denaturing at 95 °C for 5 s and annealing at 60 °C for 34 s. The relative expression of the apoptosis cytokine mRNA was measured by the 2^−ΔΔCt^ method [74]. 

### 5.8. Statistical Analysis

Data were analyzed using SPSS 17.0 software (SPSS Inc., Chicago, IL, USA, 2008), and the data of three independent experiments were expressed as mean ± standard deviation (SD). All the experimental data were analyzed for variance uniformity. When the variance was uniform, data were analyzed by a one-way ANOVA followed by Least-Significant Difference (LSD). When the variance was uneven, data were converted into logarithms, and then analyzed by one-way ANOVA followed by LSD. The significance was considered to be at the probability level of *P* < 0.05.

## Figures and Tables

**Table 1:** Primers used for qRT-PCR.

Genes	Accession Number	Orientation	Sequences (5′→3′)	Fragments Size (bp)	Tm (°C)
β-actin	AY550069	Forward	ATGCTTCTAGGCGGACTGT	211	58.2
		Reverse	CCATCCAACCGACTGCT		
Bcl-2	AB271960.1	Forward	GCGACTTTGCCGAGATGT	116	55.9
		Reverse	CACAATCCTCCCCCAGTTC		
Bax	XM_ 003127290.3	Forward	TTTGCTTCAGGGTTTCATCC	113	54.4
		Reverse	GACACTCGCTCAACTTCTTGG		
Cyto c	NM_ 001129970.1	Forward	CTCTTACACAGATGCCAACAA	139	56.1
		Reverse	TTCCCTTTCTCCCTTCTTCT		
Caspase-9	XM_ 013998997.1	Forward	GGACATTGGTTCTGGAGGATT	116	52.3
		Reverse	TGTTGATGATGAGGCAGTGG		
Caspase-3	NM_ 214131.1	Forward	GACACTCGCTCAACTTCTTGG	121	54.5
		Reverse	TTGGACTGTGGGATTGAGAC		
